# Role of the Insulin-like Growth Factor System in Neurodegenerative Disease

**DOI:** 10.3390/ijms25084512

**Published:** 2024-04-20

**Authors:** Moira S. Lewitt, Gary W. Boyd

**Affiliations:** 1 School of Health and Life Sciences, University of the West of Scotland, Paisley PA1 2BE, UK; 2 School of Health and Life Sciences, University of the West of Scotland, Hamilton G72 0LH, UK; gary.boyd@uws.ac.uk

**Keywords:** aging, Alzheimer’s disease, diabetes mellitus, insulin-like growth factor, insulin resistance, neurodegenerative disease, obesity, Parkinson’s disease

## Abstract

The insulin-like growth factor (IGF) system has paracrine and endocrine roles in the central nervous system. There is evidence that IGF signalling pathways have roles in the pathophysiology of neurodegenerative disease. This review focusses on Alzheimer’s disease and Parkinson’s disease, the two most common neurodegenerative disorders that are increasing in prevalence globally in relation to the aging population and the increasing prevalence of obesity and type 2 diabetes. Rodent models used in the study of the molecular pathways involved in neurodegeneration are described. However, currently, no animal model fully replicates these diseases. Mice with triple mutations in *APP*, *PSEN* and *MAPT* show promise as models for the testing of novel Alzheimer’s therapies. While a causal relationship is not proven, the fact that age, obesity and T2D are risk factors in both strengthens the case for the involvement of the IGF system in these disorders. The IGF system is an attractive target for new approaches to management; however, there are gaps in our understanding that first need to be addressed. These include a focus beyond IGF-I on other members of the IGF system, including IGF-II, IGF-binding proteins and the type 2 IGF receptor.

## 1. Introduction

Neurodegenerative diseases are increasing in prevalence. Understanding their pathophysiology is key to early diagnosis and identifying preventative and therapeutic approaches. Alzheimer’s disease (AD) and Parkinson’s disease (PD) are the two most common neurodegenerative disorders [1]. While the disorders are distinct, there are overlapping features: the incidence rates increase with age and are associated with cognitive impairment and loss of olfactory function. In both, there is an association between obesity and type 2 diabetes (T2D), and disease risk. There is also evidence for the role of the insulin-like growth factor (IGF) system underpinning these associations.

The aim of this review is to scope out recent advances in the field, with a focus on the IGF system in these neurodegenerative diseases. We build on the findings of an earlier review [2] of the role of the IGFs in the nervous system, focussing on recent literature by searching PubMed from 1 January 2018 to 12 February 2024 using the terms “IGF” AND “Alzheimer*” OR “Parkinson*”. References within the articles obtained by these methods were also used to retrieve key papers. The earlier article [2] reviewed the role of the IGF and IGF-binding proteins (IGFBPs) in the nervous system more generally. While that article provides a wider perspective, relevant research prior to 2018 in relation to AD and PD is also included here. 

An overview of the normal physiology of IGFs in the nervous system will be presented first, followed by an update on their role in AD and PD. Since age and dysmetabolism are common risk factors for each of these, the question of whether the IGF system is a common link will then be discussed. Finally, we highlight gaps in the literature and make recommendations for future research.

## 2. Insulin-like Growth Factor System and the Brain

The IGF system comprises an array of ligands, binding proteins, and receptors [3]. IGF-I and IGF-II have similar folded structures to proinsulin; however, the IGFs do not undergo internal processing in the way that proinsulin does to produce insulin and C-peptide. IGFs and insulin are ligands for cell-surface type 1 IGF receptor (IGF1R) and insulin receptors (isoforms IRA and IRB), all of which can form heterodimers with interrelated signalling pathways through phosphorylation of insulin receptor substrate-1 (IRS-1) and subsequently protein kinase B (Akt) and phosphatidylinositol 3-kinase (PI3K) activation. IGF function depends on the tissue expression of these receptors in different cell types and tissues, as well as the IGFBP milieu, including the presence of a soluble form of the type 2 IGF receptor (IGF2R), a monomeric receptor that has a role in internalising IGF-II and not IGF-I, as well as lysosomal enzymes. Also known as the mannose 6-phosphate receptor, IGF2R is widely distributed in the brain [4]. 

**Figure 1:**
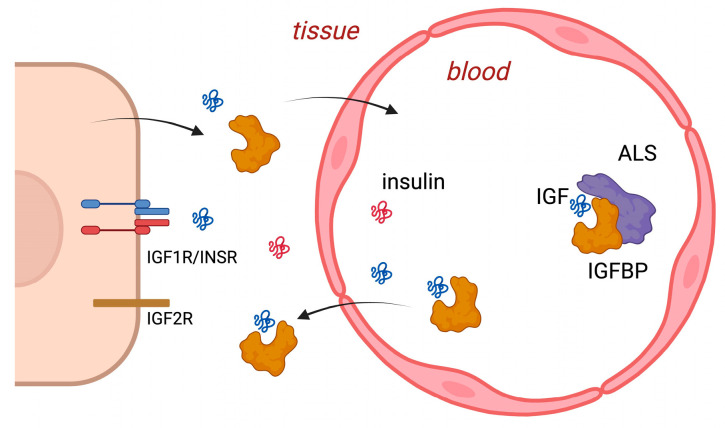
The IGF system in the circulation and peripheral tissues. IGF-I and IGF-II cross the endothelial barrier alone or in binary complexes with IGF-binding proteins (IGFBPs). IGFs associated with IGFBP-3 or IGFBP-5 can form ternary complexes with an acid-labile subunit (ALS) that are retained in the circulation. IGFs and insulin interact with cell surface type 1 IGF receptors (IGF1R) and insulin receptors (INSR) that can form heterodimers. Created with Biorender.com.

Ubiquitously expressed, tissue IGFs are derived from both endocrine and paracrine sources [5]. The endocrine role of these (~7 kDa) proteins depends on their availability in the circulation, where they associate in binary forms with IGFBP-1 to -6 (~25–45 kDa) that can cross the endothelium or tertiary forms (~140 kDa) with IGFBP-3 or -5, together with an acid-labile subunit (ALS) that is retained in the circulation (see Figure 1). Systemic circulating IGFs are available to the central nervous system by crossing fenestrated and sinusoidal capillaries, including those of the anterior pituitary, where they inhibit growth-hormone-releasing hormone (GHRH)-stimulated GH release, as well as the blood–brain barrier (BBB) [6] to directly inhibit hypothalamic GHRH secretion and other actions. While initially controversial, there is now a good understanding of the mechanisms by which peptides cross the BBB [7,8]. IGF-I not associated with IGFBPs crosses the BBB via interaction with the endothelial transporter lipoprotein-related receptor (LRP)-1 [9], independently of IGF receptors. This interaction is enhanced by neuronal activity in relation to vasodilatation.

IGF-I is expressed in neurons, particularly proliferating precursors, as well as glial cells, in a brain-region-specific manner. Along with endocrine IGF-I, these sources are likely to have neuroprotective paracrine/autocrine actions [10]. GH crosses the BBB, mostly by passive diffusion [11], and may regulate nervous system IGF-I expression [12]. Patterns of expression, and roles of IR and IGF-IR in the brain are cell-specific and context-dependent [13]. Although less well studied, it is likely that IGFBPs are also differentially expressed in a cell- and region-specific manner. The multiple actions of IGF-I from paracrine as well as endocrine sources involve interaction with other growth factor pathways, as previously reviewed [2]. IGF-I interacts with sex steroid pathways to stimulate neurogenesis in the dentate gyrus of the hippocampus and with epidermal growth factor in astroglial cell proliferation, for example. IGF-I is cleaved in the brain, and in the circulation, to form N-terminally truncated IGF-I and a glycine-proline-glutamate (GPE) tripeptide. This cleavage is inhibited when IGF-I is associated with IGFBPs. Although GPE is cleared rapidly from the circulation, it crosses the BBB and has a longer half-life in the brain where it has neuroprotective effects, a property that is being explored for the management of brain injury [14] and as a diagnostic tool in PD [15]. The role of IGF-II in the brain is less well understood. Although local mRNA expression is low, the protein is present, and it has been shown to have neuroprotective actions [16].

Conclusions relating to the expression and role of IGF system proteins in the brain rely on studies in rodent models. These, however, may not be suitable models for the study of human neurodegenerative disease. There are important differences; for example, there is more pronounced gender dimorphism in relation to insulin resistance in rodents. Cardiometabolic diseases are in low prevalence in rodents compared to humans, and studies rely on inducing disease, e.g., transgenic models or nutritional manipulation. Cell studies also have limitations as models of disease. In particular, the complexity of the tissue environment is not reflected in the influence of important regulators of IGF activity, including IGFBPs and IGFBP proteases, which are notably lacking. No animal model fully replicates human AD or PD, but they are used to identify potential molecular pathways and test potential therapies, as reviewed below.

## 3. Alzheimer’s Disease (AD)

**Figure 2:**
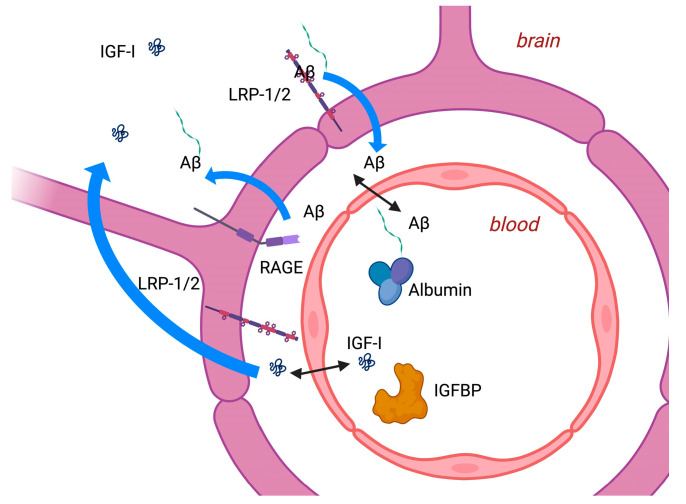
Transport of β-amyloid (Aβ) and insulin-like growth factor (IGF)-I at the blood–brain barrier involves interaction with lipoprotein receptor 1 and 2 (LRP1/2) and the receptor for advanced glycation end products (RAGE). Aβ associates with carrier proteins such as albumin and IGF-I with IGF-binding proteins (IGFBPs). Created with Biorender.com.

Typically, AD is characterised by memory loss, usually associated with a decline in visuospatial and executive functions [1]. There are two neuropathological hallmarks of AD: neuronal plaques of abnormal β-amyloid (Aβ) accumulation extracellularly and fibrillary tangles composed of intracellular accumulations of hyperphosphorylated microtubule associated protein tau (MAPT). Accumulation of these proteins is associated with microglial cell activation and a pro-inflammatory environment, culminating in hippocampal and cortical neurodegeneration. Oligomeric forms of Aβ are likely to be deposited and lead to neurotoxicity, perhaps by impairing insulin/IGF signalling, while soluble monomeric Aβ molecules are associated with activation of insulin/IGF pathways [17] with multiple effects, some of which lead to neuronal rescue but which also promote further Aβ accumulation, increasing secretion, and reducing degradation. Aβ levels represent a balance between Aβ production and clearance. Aβ movement across the BBB is bidirectional, with LRP-1 transporting Aβ out [18] and the receptor for advanced glycation end-products (RAGE) transporting into [19] the brain (see Figure 2). LRP1 also binds apolipoprotein E (APOE) forms [18]. IGF-I is said to also interact with LRP-2 [20] to facilitate Aβ transport [21,22], in part by enhancing Aβ carrier proteins such as albumin and transthyretin.

Aβ is a cleavage product of amyloid precursor protein (APP); and presenilin (PSEN)1 and PSEN2 are core proteins in the γ-secretase complex involved in this process. Mutations of the genes *APP*, *PSEN1* and *PSEN2* are implicated in familial, early onset AD, while in sporadic forms, environmental factors interact with multiple other genes, including *APOE* variants, to contribute to AD risk [23,24]. Along with *APOE* genotype, sex modifies the relationship between IGF-I and cognitive processing [25]. Females are at greater risk of AD, although women live with more disability for longer [26]. Of interest, in mice with *APP* and *PSEN1* mutations, ovariectomy exacerbates learning and memory defects, neuron loss and altered synaptic structure in the hippocampus, along with altered energy metabolism in the brain, possibly via the IGF1R/GSK-3β signalling pathway [27]. On the other hand, in a mendelian randomisation study, while IGF-I could not be excluded, higher total testosterone was associated with reduced AD risk in both men and women [28].

The presence of cerebrovascular disease, hypertension or traumatic brain injury increases the risk of Alzheimer’s and other dementias [1]. Obesity and T2D are associated with an increased risk of approximately 1.6-fold [1]. Factors that reduce the clinical risk and underpin approaches to prevention and management therefore include encouraging intellectually enriched lifestyles, exercise, and good nutrition, as well as the management of hypertension and hyperlipidemia. Of note, Aβ accumulation starts 20 years before clinical symptoms in familial AD and fewer years for sporadic AD [29]. Approaches to early diagnosis have been proposed, including positron emission tomography [1], cerebrospinal fluid (CSF) Aβ [30] and IGF-I levels [31] as well as loss of olfactory function [32]. Pharmacologic interventions currently being investigated include raising acetylcholine levels using acetylcholinesterase inhibitors (e.g., donepezil) alone or combined with suppressing glutamate-mediated excitotoxicity with *N*-methyl-D-aspartate (NMDA) receptor antagonists [1].

Since IGF-I has neuroprotective actions, it has been argued that reduced brain IGF-I receptor sensitivity and/or IGF-I deficiency underlie vulnerability to AD [29]. Unbound IGF-I is higher in AD in the brain than in control cases [33]. Higher serum IGF-I concentrations are associated with lower nuclear magnetic resonance image markers of AD as early as midlife [34]. In established AD, on the other hand, serum IGF-I does not associate with brain volumes, supporting IGF-I resistance [35]. A study, using a mendelian randomisation design, of *IGF1* and *IGFBP3* single nucleotide polymorphisms that raise serum IGF-I indicates that circulating IGF-I is not associated with AD risk [36]. Supporting the notion of IGF-I resistance, AD patients have a greater serum IGF-I response to exercise compared to older adults without AD [37], and brain cells from AD patients have reduced insulin/IGF-I signalling activity [38]. Down-regulation of IGF1R reduces Aβ deposition induced by chronic sleep restriction in mice [39]. On the other hand, tau phosphorylation is increased in *IGF1* knockout mice [40].

While no animal model fully replicates the disease, mutations in *APP*, *PSEN1* and *PSEN2,* linked to human AD, have been introduced in mice. When *APP* and *PSEN* mutations are combined, the amyloid phenotype is enhanced, with greater relative Aβ accumulation; however, tau pathology is not prominent in these models [41,42]. In mice with *APP* mutations, cortical IGF1R expression is reported to be down-regulated [43], and short-term peripheral administration of IGF-I has no effect on CSF or plasma Aβ concentrations [44]. Prenatal stress aggravates the *APP* phenotype [45]. When crossed with mice deficient for IRS-2, Aβ accumulation is delayed, and premature mortality is reversed in female mice [46]. The marked alteration in Aβ levels and histopathology in the APP model with ablation of IRS-2 is not seen with IRS-1 [47]. Neuronal IGF1R deficiency, or IR deficiency, also delays Aβ accumulation [46,48]. However, while neuronal IGF1R deficiency reverses premature mortality in both male and female mice [46], neuronal IR deficiency does not [48]. Thus, neuronal IGF-I resistance appears to be protective and linked to the processing of Aβ, with a key role for IGF1R/IRS-2. The role of IGF-II in this model is less clear; genome-wide RNA sequencing analysis has demonstrated a reduction in IGF-II expression, while targeted delivery of IGF-II to the hippocampus is associated with activated PI3K/Akt/CREB signalling and attenuated Aβ accumulation, oxidative stress and memory decline [49]. Studies using the *APP* mutation that explore the role of the IGF system are summarised in Table 1.

Double transgenic mice with *APP*/*PSEN1* mutations have evidence of IGF-I resistance, with reduced IGF1R phosphorylation and Akt response to IGF-I in hippocampal and cortical tissue slices [51]. In that study, increased IGF1R expression was demonstrated in hippocampal slices. However, reducing IGF signalling with IGF1R heterozygous knockout [52] or inhibition of IGF1R with picropodophyllin that crosses the BBB, attenuates Aβ accumulation [53] and, when *APP*/*PSEN1* mice are crossed with neuronal IGF1R knockout, Aβ accumulation and neuroinflammation are reduced and differential gene expression associated with AD is reversed [54,55]. When *APP*/*PSEN1* mice are crossed with GH-deficient Ames dwarf mice, brain IGF-I levels decrease and the AD phenotype is attenuated [56], with changes in microRNA expression predicting a functional role for the PI3K/Akt/mTOR/FOXO pathway [57]. Somewhat paradoxically, when crossed with mice with liver-specific IGF-I deficiency, and therefore reduced endocrine IGF-I, mice with *APP*/*PSEN1* mutations have earlier Aβ accumulation [58]. Furthermore, IGF-I delivered subcutaneously reduces Aβ overexpression in the cortex and hippocampus [59], increases brain Aβ complexed with carrier proteins [61], and improves behavioural patterns [60,61]. Subcutaneous IGF-I also reduces Aβ accumulation in rats administered Aβ intracerebroventricularly [66]. Central delivery of IGF-I also reduces Aβ accumulation in this model, enhances spatial learning and memory and relieves anxiety behaviour [67,68]. The retained capacity of microglia to express IGF-1 in these mice is encouraging [69]. While there is central IGF-I resistance, increasing IGF-I availability may play a role in reducing Aβ accumulation. Consistent with this, IGF-I and insulin, via PI3K/Akt activated pathways, have been shown to reduce APP phosphorylation in primary cortical neurons [70]. Treatment of *APP*/*PSEN1* mice with recombinant choline acetyltransferase or donepezil improved the AD phenotype, with the former implicated in neuroprotection, synaptic plasticity, neuronal survival and cerebrovascular remodelling, and the latter in altering the immune inflammatory response and insulin/IGF signalling [71].

Tau accumulation and neurodegeneration are not prominent in the mutant *APP* and/or *PSEN* models described above. Neuronal tau inclusions with neurodegeneration are present with transgenic overexpression of *MAPT* mutations in mice, with line P301L, expressing all the tau isoforms on a background of mouse *MAPT* knockout and having neurofibrillary tangles [46]. In this model, IGFBP-2, whose CSF levels are associated with tau pathology, is differentially expressed compared to the wild-type mice [72]. When P301L is co-expressed with mutant *APP* and/or *PSEN*, this line is most relevant to human disease [41]. However, studies of the impact on IGF1R expression and IGF-I resistance are lacking. P301L expressed in a triple mutant with *APP* and *PSEN* mutations (3xTg-AD) has been used to explore the effect of allopregnanolone, an endogenous neurosteroid; regeneration of grey and white matter in response to allopregnanolone is associated with increased neuronal IGF-I and IGF1R expression [64]. Protein restriction attenuates the AD phenotype in these mice [63]. Regular resistance training reduces Aβ accumulation and increases IGF-I in the hippocampus of 3xTgAD mice [65], and photobiomodulation therapy upregulates TGFβ1/IGF-1/BDNF [73]. Maternal 3xTgAD mice exposed to gestational environment enrichment have activated IGF1R/CaMKIV/ HAT/BDNF signalling and preserved synaptic plasticity and memory capacity, ameliorating AD pathology [74]. When germ-free 3xTgAD mice are recolonised with gut microbiota from human AD patients, AD pathologies are exacerbated [75].

## 4. Parkinson’s Disease (PD)

The pathological hallmark of PD is insoluble aggregates of misfolded α-synuclein (Lewy bodies) in the cytoplasm of dopaminergic neurons of the substantia nigra pars compacta region, leading to neuronal loss [1,76]. The cardinal features of PD include bradykinesia, resting tremor, rigidity and postural instability, which are the sole basis for diagnosis [1]. Pharmacologic therapies target these motor features, enhancing dopamine signalling. Lewy bodies are found throughout the central nervous system, and non-motor symptoms may precede the motor features by many years. Cognitive decline occurs more commonly in those with late-onset disease, along with postural instability and gait disturbances. PD is one of a range of α-synuclein pathologies. There is overlap between PD with dementia and the clinical entity dementia with Lewy bodies (DLB), which is the second most common form of dementia, in which cognitive impairment occurs early and usually precedes motor symptoms [1]. Both are characterised by α-synuclein and ubiquitin aggregation. Other non-motor features in PD include dysautonomia and anosmia. The risk of PD is higher in men, and women are reported to present with milder symptoms and have a slower progression [1]. There is evidence that dopaminergic protection by oestrogens involves PI3K signalling pathway activation [76]. Alongside male sex, elevated serum IGF-I is associated with increased PD risk [77], and IGF-I levels correlate with cognitive dysfunction [78]. On the other hand, circulating IGF-I levels increase alongside improved motor function in relation to pharmacologic therapy for PD [79]. There is evidence of insulin/IGF-I resistance in nigral dopaminergic neurons, with increased phosphorylated IRS1 in post-mortem samples from humans [80].

Most cases of PD are sporadic; however, several genes have been identified as monogenic forms, including mutations in *Parkin* and *Pink*-1 that cause autosomal recessive early onset PD, and *LRRK2*, which manifests as a benign tremor (asymmetric PD), along with an increased risk of cognitive and olfactory dysfunction [1]. Human carriers of the *LRRK2* mutation have reduced IGF2R [81] and, although knock-out or knock-in rodent models do not have a PD phenotype, changes in IGF2R localization are observed [82]. Cells derived from *LRRK2* knock-down mice and knock-out rats have reduced insulin-dependent translocation of glucose transporter type 4 [83].

When mutated human α-synuclein is introduced with a recombinant adeno-associated virus vector into rats, there is a similar pattern to that in humans of phosphorylated IRS-1 expression in nigral dopaminergic neurons [80]. Grb10-interacting GYF Protein 2 (GIGYF2) binds activated IGF-I and insulin receptors and maps to a region linked to familial PD. GIGYF2 heterozygous mice (+/−) exhibit motor dysfunction; however, they have brainstem and cerebellum, but not substantia nigra, α-synuclein-positive neuritic plaques [84].

Many animal models rely on neurotoxins that cause lesions of the dopaminergic neurons of the substantia nigra, e.g., 1-methyl-4-phenyl-1,2,3,6-tetrahydropyridine (MPTP). When MPTP is administered to mice, transcriptomic profiling of differentially expressed genes in the striatum region suggests IGF-I might play a key role in PD development [85]. When MPTP is administered to IGF1R heterozygote knockout mice, oxidative stress-associated genes are down-regulated, and there is a reduced neuro-inflammatory response compared to wild-type mice [86]. In this model, there is evidence that the G protein-coupled estrogen receptor is involved in the neuroprotective effect of IGF-I through PI3K signalling [87].

## 5. Risk Factors for Neurodegenerative Disease

Clinical and pathological features are shared between AD and PD [1]: both are associated with cognitive decline, depression/anxiety and olfactory dysfunction; along with protein misfolding and aggregation, and activation of inflammation, with proinflammatory mediators and reactive oxygen species (ROS) contributing to a vicious cycle of neuronal cell death. While a causal relationship is not proven, the fact that age, obesity and T2D are risk factors in both strengthens the case for the involvement of the IGF system in these disorders.

### 5.1. Aging

Aging is the leading risk factor for neurodegenerative diseases [88]. The incidence of sporadic AD increases exponentially with age, from a yearly risk of approximately 0.5% at the age of 65–70, to more than 6% over the age of 85 [23]. In PD, the prevalence is approximately 1% over the age of 60 and 3% over the age of 80 [1]. This association between age and disease prevalence underpins the speculation that the IGF system is involved. The insulin/IGF signalling pathway is evolutionarily conserved from yeast to mammals and is a central modulator of metabolic pathways that are linked to aging, mediating in part the positive impact of caloric restriction and physical exercise on brain aging [88,89]. While it has a key role in longevity, both neuroprotective and detrimental neurological effects have been reported. In humans, IGF polymorphisms associated with lower circulating IGF-I concentrations are associated with longevity [90]; yet, paradoxically, a lower IGF-I level is considered a biomarker for frailty [91]. We have previously suggested that there is a U-shaped relationship between IGF-I concentrations and cognitive function [2], with the lowest and highest IGF-I levels associated with poorer cognitive function. Circulating IGF-I concentration is not necessarily useful as a marker of age-related cognitive decline [92].

Studies in Caenorhabditis elegans highlight a lack of overlap between longer lifespan and healthy life expectancy and the importance of considering tissue-specific changes in the aging process [93]. Studies in this model indicate that the insulin/IGF pathway links the onset of toxic protein aggregation to aging [94]. Studies in mice indicate that there is reduced brain IGF-I function during aging [95] and that, under these circumstances, preserved endocrine IGF-I reaching the brain might be essential to resilience to neurodegenerative disease. In humans, higher ‘free’ circulating IGF-I concentrations in mid-life are associated with reduced cognitive decline in late-life [96] and reduced risk of AD [97]. It has been proposed that, in the absence of IGF-I, ligand-independent IGF1R activity is unchecked and potentially detrimental [98]. However, other studies suggest that insulin/IGF signalling pathways protect against proteotoxicity associated with aging. Mice with GH deficiency or GH resistance, and low circulating IGF-I live longer [99]. However, these mice have some tissue IGF-I, including the brain. In aged mice, local delivery of IGF-I to the basal forebrain increases neuronal activity and sensory processing; and improves whisker response when delivered directly to the primary somatosensory cortex [100]. 

### 5.2. Dysmetabolism

The brain is highly dependent on glucose as a source of energy; it also plays a role in glucose homeostasis [101]. Insulin crosses the BBB via a unidirectional, blood-to-brain saturable transporter [102], and it is likely that most insulin acting in the brain is derived from endocrine sources [7,103]. Neuron-specific *LRP1* knockout mice have impaired insulin signalling and glucose intolerance in the brain [104]. Brain insulin/IGF signalling is also important to whole-body metabolism. Neuron-specific IR-knockout mice have normal development and normal brain size but mild peripheral insulin resistance [105]. While this may be due to the paracrine action of insulin, which is expressed in the brain [106], it is perhaps more likely to be derived from endocrine sources. IGF-I increases insulin sensitivity via a post-receptor effect [107], therefore changes in insulin action may be due to altered IGF-signalling through the IR.

Metabolic syndrome components are associated with a predisposition to clinically significant neurodegenerative disease; for example, there are associations between obesity and/or T2D and both AD [108,109] and PD [110,111]. Shared pathological features include mitochondrial dysfunction, oxidative stress, and insulin resistance, along with the exacerbation of abnormalities of lipid metabolism [111,112,113]. Drugs targeting these pathways are therefore being considered in the management of AD and PD [114,115,116,117]. Protein expression in mice expressing human Aβ1-42 from a transgene shows overlapping expression patterns with diet-induced obesity, with effects on proteostasis, apoptosis and synaptic vesicles [118]. When *APP* mutations are expressed in obese mice with NPY diabetes, the AD phenotype is enhanced [50].

In the absence of T2D, AD is associated with reduced brain glucose uptake and utilisation, as well as brain insulin resistance; it has been labelled Type 3 diabetes mellitus [119,120]. There is also evidence of insulin resistance in the brains of people with PD without T2D [111]. Brain insulin resistance is an early feature in AD and is associated with IGF-I resistance and IRS-1 dysfunction [121], the features of which in animal models are described above. When transgenic mice with combined *APP*/*PSEN1* mutations are crossed with mice with hyperglycaemia induced by IGF-II overexpression in the pancreas, insulin resistance is enhanced [62]. This supports the notion that AD is a risk factor for T2D. In this model, mice on high-fat diets had greater cognitive impairment, suggesting that dietary choice on the background of T2D might influence the risk of AD. 3xTg-AD transgenic mice have a similar phenotype to the intracerebroventricular administration of streptozotocin [122]. Insulin, delivered subcutaneously, decreases Aβ deposition and improves memory in 3xTgAD mice fed a high fat diet [123]. Insulin delivered intranasally has been reported to improve cognition in this model [124]. 

## 6. Conclusions

Recent research has started to unravel the paradox of insulin/IGF signalling having both neuroprotective and neurotoxic actions in healthy aging and degenerative disease. Further understanding of the relative roles of endocrine and paracrine IGFs and insulin will be important in the development of new therapeutics and in identifying how they might be targeted effectively to relevant tissue. Notably, there has been little progress in the areas that we recommended in our previous review [2]. There remain gaps in the literature in relation to the role of IGF-II and the IGFBPs. Since there is increasing evidence of the association between insulin resistance and neurodegenerative disease, the role of IGF-II, which has a higher affinity than IGF-I for IRA/IGF1R hybrids [125] and interacts with IGF2R, including its soluble form, is recommended as a research focus. The patterns of IGF-II expression differ between humans and rodents, and alternative models should be explored; however, it should be noted that it is expressed at high levels in adult rat brains, compared with other tissues [126]. The IGFBPs are important regulators of IGF action, and they also have IGF-independent actions [3]. Therefore, in addition to studies of local expression, their actions in the brain are worth delineating.

Insulin, delivered intranasally, has been reported to improve cognition in an animal model [124]; the use of IGF as an insulin sensitiser, alone or combined with insulin, would be worthwhile. The spectrum of IGF1R actions in the aging brain has been highlighted [98]. We suggest that exploration of the mechanisms underlying IGF1R signalling that are independent of IGF-I action and reduce resilience to neurodegenerative disease with age might also identify novel therapeutic approaches. This would include the interaction with other signalling pathways and, therefore, the potential for a combination approach to therapy.

## Figures and Tables

**Table 1:** Impact of changes in insulin/IGF availability in mouse models of Alzheimer’s diseases.

AD Mutation	IGF1R Expression	IGF-I Resistance	+IGF Animal Model	AD Phenotype	References
*APP*	reduced	increased			[43,44]
			× *IRS2*−/−	attenuated	[47]
			× *IRS1*−/−	no change	[47]
			× neuronal *IGF1R*−/−	attenuated	[46]
			× neuronal *IR*−/−	attenuated	[48]
			hippocampal IGF-II delivery	attenuated	[49]
			× *ob/ob* obese	enhanced	[50]
			× NPY diabetes	enhanced	[50]
*APP* + *PSEN*	increased	increased			[51]
			× *IGF1R*+/−	attenuated	[52]
			IGF1R inhibitor	attenuated	[53]
			× neuronal *IGF1R*−/−	attenuated	[54,55]
			× GH deficiency (Ames)	attenuated	[56,57]
			× hepatic *IGF1*−/−	enhanced	[58]
			IGF-I delivery peripherally	attenuated	[59,60,61]
			× pancreatic *IGF2*+	enhanced	[62]
*APP* + *PSEN* + *MAPT*	not determined	not determined			
			protein restriction (↓ IGF-I)	attenuated	[63]
			allopregnanolone (↑ IGF1R)	attenuated	[64]
			resistance exercise (↑ IGF-I)	attenuated	[65]

## Data Availability

No new data were created or analysed in this study. Data sharing is not applicable to this article.
